# Reproducibility of tract‐based white matter microstructural measures using the ENIGMA‐DTI protocol

**DOI:** 10.1002/brb3.615

**Published:** 2017-01-14

**Authors:** Ashley Acheson, S. Andrea Wijtenburg, Laura M. Rowland, Anderson Winkler, Charles W. Mathias, L. Elliot Hong, Neda Jahanshad, Binish Patel, Paul M. Thompson, Stephen A. McGuire, Paul M. Sherman, Peter Kochunov, Donald M. Dougherty

**Affiliations:** ^1^ Department of PsychiatryUniversity of Texas Health Science Center at San AntonioSan AntonioTXUSA; ^2^ Research Imaging InstituteUniversity of Texas Health Science Center at San AntonioSan AntonioTXUSA; ^3^ Department of PsychiatryMaryland Psychiatric Research CenterUniversity of Maryland School of MedicineBaltimoreMDUSA; ^4^ Russell H. Morgan Department of Radiology and Radiological ScienceJohns Hopkins UniversityBaltimoreMDUSA; ^5^ Oxford Centre for Functional MRI of the BrainUniversity of OxfordOxfordUK; ^6^ Department of PsychiatryYale University School of MedicineNew HavenCTUSA; ^7^ Imaging Genetics Center, Stevens Neuroimaging and Informatics InstituteKeck School of Medicine of USCMarina del ReyCAUSA; ^8^ Department of Neurology59th Medical WingLackland AFBTXUSA; ^9^ Department of Neuroradiology59th Medical WingLackland AFBTXUSA

**Keywords:** diffusion tensor imaging, ENIGMA‐DTI, fractional anisotropy, reproducibility, white matter microstructure

## Abstract

**Background:**

In preparation for longitudinal analyses of white matter development in youths with family histories of substance use disorders (FH+) or without such histories (FH−), we examined the reproducibility and reliability of global and regional measures of fractional anisotropy (FA) values, measured using the Enhancing Neuro Imaging Genetics Through Meta Analysis (ENIGMA)‐diffusion tensor imaging (DTI) protocol. Highly reliable measures are necessary to detect any subtle differences in brain development.

**Methods:**

First, we analyzed reproducibility data in a sample of 12 healthy young adults (ages 20–28) imaged three times within a week. Next, we calculated the same metrics in data collected 1‐year apart in the sample of 68 FH+ and 21 FH− adolescents. This is a timeframe where within subject changes in white matter microstructure are small compared to between subject variance. Reproducibility was estimated by examining mean coefficients of variation (MCV), mean absolute differences (MAD), and intraclass correlations (ICC) for global and tract‐specific FA values.

**Results:**

We found excellent reproducibility for whole‐brain DTI‐FA values and most of the white matter tracts, except for the corticospinal tract and the fornix in both adults and youths. There was no significant effect of FH‐group on reproducibility (*p *=* *.4). Reproducibility metrics were not significantly different between adolescents and adults (all *p *> .2). In post hoc analyses, the reproducibility metrics for regional FA values showed a strong positive correlation (*r *= .6) with the regional FA heritability measures previously reported by ENIGMA‐DTI.

**Conclusion:**

Overall, this study demonstrated an excellent reproducibility of ENIGMA‐DTI FA, positing it as viable analysis tools for longitudinal studies and other protocols that repeatedly assess white matter microstructure.

## 1 Introduction

Adolescence is a crucial period for brain development and one key developmental process occurring is the maturation of cerebral white matter, including the continual myelination of axons, facilitating more efficient neural functioning as youths reach adulthood (Bartzokis et al., 2010; Gogtay & Thompson, 2009; Gogtay et al., 2004; Kochunov, Glahn, et al., 2011). One widely used noninvasive method to assess the development of white matter microstructure is diffusion tensor imaging (DTI), a variant of MRI. The most commonly used index of white matter microstructure derived from DTI is fractional anisotropy (FA), which is a sensitive, quantitative measure of white matter microstructure (Basser, Mattiello, & LeBihan, 1994; Basser & Pierpaoli, 1996) used to index white matter microstructure changes across the lifespan (Hasan et al., 2009; Karlsgodt et al., 2014; Kochunov et al., 2015; Kochunov, Glahn, et al., 2011). FA also serves as an endophenotype for many common neurological and psychiatric disorders (Barysheva, Jahanshad, Foland‐Ross, Altshuler, & Thompson, 2013; Carballedo et al., 2012; Clerx, Visser, Verhey, & Aalten, 2012; Kochunov, Williamson, et al., 2016; Mandl et al., 2012; Sprooten et al., 2004; Teipel et al., 2011). Here, we evaluate the reproducibility of FA values generated from a novel, standardized DTI protocol developed by the ENIGMA‐DTI working group to extract DTI phenotypes. The ENIGMA‐DTI protocol was specifically developed and validated to provide global and regional measurements with replicable heritability patterns across diverse study populations and neuroimaging protocols (Jahanshad et al., 2013; Kochunov et al., 2015; Kochunov et al., 2013). The ENIGMA‐DTI protocol is based on the popular tract‐based spatial statistics approach and uses a custom minimal deformation template based on data from multiple sites to take into account site differences and define a population‐wide skeleton in an atlas space that includes major white matter tracts.

Using this standard and validated DTI analysis protocol, we previously reported that youths and young adults with family histories of substance use disorders (FH+) had decreased FA in frontostriatal and frontocortical white matter tracts relative to those without such histories (FH−), prior to the onset of any regular substance use (Acheson, Wijtenburg, Rowland, Bray, et al., 2014a; Acheson, Wijtenburg, Rowland, Bray, Winkler, et al., 2014b). This suggests that FH+ individuals, who are at increased risk for developing substance use disorders themselves, have white matter microstructural impairments that predate substance abuse and may contribute to their elevated risk. As part of this longitudinal study, we perform annual assessments to map how white matter development across adolescence is influenced by family history as well as the initiation and progression of problem substance use. FA values generated from the ENIGMA‐DTI protocol must therefore be sufficiently reproducible and stable to support the longitudinal analyses and ensure appropriate sensitivity to detect the FH‐related differences in the adolescent development.

We examined reproducibility estimates of FA values generated from a group of healthy adults imaged three times in 1 week and compared those estimates to reproducibility estimates in the youth cohort imaged 1‐year apart. The annual rate of change in FA values during adolescent development are expected to be small (~ 10^−3^ FA units/year) compared to the variability among subjects (Kochunov, Glahn, et al., 2011). Therefore, in order to be able to pick up group differences between adolescents developing under different conditions, we need to ensure the measurements we evaluate are reliable and reproducible. Here, the data from the two groups of subjects can be used to answer the following three scientific questions:

1. Can we consistently measure global and regional FA values in the same subjects within a relatively small (1‐year) developmental interval?
2. Are estimates of reproducibility in the adolescent cohort comparable to those in healthy adults where no change in FA is expected?
3. Do measurements of reproducibility measured in two groups explain the by‐tract variance in additive genetic variations previously reported by ENIGMA‐DTI in the largest international heritability study of DTI‐FA values to date (Kochunov et al., 2015)? If a measure is highly reliable and reproducible, we expect that a greater component of the variability due to genetics may be detected, and lower reproducibility measurements for regional FA values may be responsible for lower heritability estimates in certain tracts.

There is no single, established metric for evaluating reproducibility in neuroimaging studies. We calculated three statistical measurements of reproducibility, commonly used in neuroimaging studies: the mean absolute differences (MAD), the mean coefficients of variation (MCV), and the intraclass correlations (ICC). The MAD is the absolute difference between first and second visit and provides assessments of the absolute difference between two datasets. The MCV is calculated as the standard deviation normalized by the average for two visits and provides a general assessment of variance in each dataset relative to the mean. The ICC is a form of correlation analysis that assesses the consistency of variability among subjects on two visits. Thus, using all three metrics enables a thorough assessment of reproducibility.

These three metrics were applied to the global and regional FA values in 12 healthy young adults (ages 20–28 years) imaged three times within a week to estimate variability in regional FA values to examine the stability of the technique. The reproducibility assessed in the healthy adults, within whom no change is expected in the given interval, will help us interpret our longitudinal assessment in the adolescent population. To examine the variability between time points in the adolescent cohort, we also calculated MAD, MCV, and ICC.

## 2 Methods

### 2.1 Participants

Twelve healthy young adults (ages = 20–28) were imaged three times within 1 week using the same protocol to calculate an estimate of the variance in FA values over this time period. These estimates of variance were also examined in 89 children and adolescents (10–14 years old at first scan, average age = 12.9 ± 1.0 years, *N *= 21/68 FH−/+) from an ongoing longitudinal study of adolescent development and substance use involvement in youths at elevated risk for substance use disorders (Acheson, Wijtenburg, Rowland, Winkler, et al., 2014b; Dougherty et al., 2014; Ryan et al., 1995). All participants signed consent forms approved by the Institutional Review Board of the University of Texas Health Science Center at San Antonio, which approved the study procedures. In addition, privacy was further protected by a Certificate of Confidentiality from the Department of Health and Human Services.

### 2.2 Family history of substance use disorders

Family history classification for the youth cohort was established using the Family History Assessment Module (Rice et al., 2012) based on the parent report. All FH+ participants had a biological father with a past or present substance use disorder.

### 2.3 Collection and processing of magnetic resonance imaging data

All MRI procedures were performed at the Research Imaging Institute, University of Texas Health Science Center at San Antonio using the same Siemens Tim Trio 3T MR system (Erlangen, Germany) equipped with a multichannel head coil using a protocol described elsewhere (Wijtenburg et al., 2010). Imaging sessions were conducted approximately 1‐year apart (±30 days) for the youth cohort and 24–48 hr apart for the adult cohort. A single‐shot, echo‐planar, single refocusing spin‐echo sequence was used to acquire diffusion‐weighted data with a spatial resolution of 1.7 × 1.7 × 3.0 mm. The sequence parameters were as follows: TE/TR = 83/7000 ms, FOV = 200 mm, two diffusion‐weighting values, *b *=* *0 and 700 s/mm^2^, and five *b*_0_ (nondiffusion‐weighted) images, 64 isotropically distributed diffusion‐weighted directions, and axial slice orientation with 50 slices and no gaps. The number of directions, *b*_*0*_ images, and the magnitude of the *b* values were calculated using an optimization technique that maximizes the contrast to noise ratio based on the average diffusivity of the cerebral white matter and the T_2_ relaxation times (Jones, Horsfield, & Simmons, 2007). High‐resolution T1‐weighted data were also collected using an optimized protocol described previously (Kochunov et al., 1999).

### 2.4 Extraction of whole‐brain average and tract‐based FA values

ENIGMA‐DTI (RRID:SCR_014649) protocols were used to extract whole‐brain and tract‐wise average FA values for both datasets. These protocols are detailed elsewhere (Jahanshad et al., 2013) and are available online, at http://www.nitrc.org/projects/enigma_dti. Briefly, FA images from subjects were nonlinearly registered to the ENIGMA‐DTI target FA image using FSL (RRID:SCR_002823)'s FNIRT (Smith et al., 2006). This target was created as a “minimal deformation target” based on images from the participating studies as described previously (Jahanshad et al., 2013; Kochunov, Lancaster, & Fox, 2014; Kochunov et al., 2006). The data were then processed using FSL's tract‐based spatial statistics (TBSS; http://fsl.fmrib.ox.ac.uk/fsl/fslwiki/TBSS) analytic method (Smith et al., 2016) modified to project individual FA values onto the ENIGMA‐DTI skeleton. After extracting the skeletonized white matter and the projection of individual FA values, ENIGMA tract‐wise regions of interest (ROIs), derived from the Johns Hopkins University (JHU) white matter parcellation atlas (Mori et al., 2008), were transferred to extract the mean FA across the full skeleton and average FA values for 18 major white matter tracts (Table S1). The whole‐brain average FA values were calculated to include all voxels in the ENIGMA‐DTI skeleton. The protocol, ENIGMA‐DTI template FA image along with its skeleton and mask, source code and executables, are all publicly available. (http://www.nitrc.org/projects/enigma_dti).

### 2.5 Quality assurance/quality control (QA/QC)

Quality assurance and quality control QA/QC was conducted according to the ENIGMA‐DTI protocol (http://enigma.usc.edu/protocols/dti-protocols/). QA/QC checks consisted of inspecting the data, vector gradients, registration, and average skeleton projection distance. The data were visually inspected during MRI session to eliminate obvious image distortions due to severe motion. If excessive blurring, data distortions, or loss of SNR were observed during the scan acquisition, the study was interrupted, subjects were instructed to keep still, and the DTI sequence was repeated.

For data without gross motion artifacts, the average estimate of subjects’ motion was calculated by spatially registering consequent DTI images and calculating the root‐mean‐square (RMS) differences between individual transformation matrices and a unit matrix using the RMSDIFF program distributed with FSL. The identity matrix, where diagonal elements are set to 1 and all others set to 0, indicates the case of no difference indicating no interscan motion. The RMSDIFF program reports an RMS difference (in mm) between two matrices: larger values indicate greater differences between the alignment matrix and the identity matrix. This number is sensitive to both translations and rotations, providing a good estimate of the motion. The average estimate per subject is obtained by averaging RMS distance for individual volumes.

The average projection distance is another QA measurement proposed by the ENIGMA‐DTI workgroup. This measure assesses the registration quality between individual images and the ENIGMA‐DTI template by calculating the average projection distance for the group‐wide skeleton. The skeleton projection step corrects for minor spatial mismatch between the centers of the track on the template versus the individual image. A higher projection distance may indicate problems with aligning individual brain to the template. The ENIGMA‐DTI protocol currently specifies no critical cutoff for the projection distance, and average projection distance for ENIGMA studies of 3.6 ± 0.2 mm. However, in this study, we repeated DTI scans for sessions with projection distances that exceeded 3.8 mm.

### 2.6 Statistics

Average motion per diffusion‐weighted volume and the average projection distances between the three groups were assessed with one‐way ANOVAs (*p *= .05/18, Bonferroni correction threshold for multiple comparisons). For the healthy young adult cohort, reproducibility between visits for 18 tracts was assessed via mean coefficients of variation (MCV), mean absolute differences (MAD), and intraclass correlations (ICC). Calculations were performed using SPSS (Version 23, RRID:SCR_002865). To estimate differences in variance over one year, these same metrics were calculate in the FH+ and FH− cohorts, separately as well as in both groups combined. t‐tests were performed to assess differences between MCV and MAD between FH+ and FH− with significance set at *p *= .0028 (*p *= .05/18). t‐tests were also performed to ensure that the subjects in the FH+ and FH− groups were not significantly different in terms of age or time between scans. Chi‐squared tests were performed to ensure they were not significantly different based on measures of sex, race, or ethnicity. To assess the differences in reproducibility measures between all three groups (young adults and FH+ and FH− youths), a one‐way ANOVA was performed with significance set to *p *= .0028 to account for multiple comparisons. Additionally, we performed a linear correlation analysis between the measurements of reproducibility calculated in the cohort of adolescents and the tract‐based estimate of heritability published by ENIGMA‐DTI study (Kochunov et al., 2015).

## 3 Results

Demographics for young adult participants in the replication study and adolescents participating in the longitudinal study are detailed in Table 1. *N *= 25 imaging sessions were repeated due to QA/QC problems identified during postprocessing. This consisted of exceeding the average motion threshold of 2.5 mm for 15 sessions and exceeding the projection distance threshold of 3.8 mm for 10 sessions. Individuals with failed QC data were rescanned within 2 weeks. All repeated sessions satisfied QA/QC requirements. For DTI data that passed QA/QC requirements, the average motion per TR was 1.18 ± 0.63 mm, and average projection distance was 3.33 ± 0.31 mm. There were no significant differences in motion estimates between the adults (1.19 ± 0.56 mm) and FH+ and FH− subjects (1.17 ± 0.61 mm vs. 1.18 ± 0.70 mm, *p *=* *.8). Likewise, there were no significant differences in the average projection distance for adults (3.20 ± 0.65 mm, *p *=* *.2) and FH+ and FH− subjects (3.32 ± 0.62 mm vs. 3.33 ± 0.67 mm, *p *=* *.7). Mean FA values for each tract for both the adults and youths are shown in Table S1. There was no significant correlation between average FA values and either average motion or projection distance (*r *<* *.05, *p *>* *.3; Figures S1 and S2). In the post hoc analysis for the 25 imaging sessions that failed the QA/QC measurements, the negative correlations between FA and average motion or projection distance were also nonsignificant (*r *=* *−.41 and −0.36, *p *>* *.1, for average motion and projection distance, respectively).

**Table 1:** Participant demographics

	Adults	FH+	FH−
Gender (M/F)	12/0	33/35	13/8
Age at first scan (years) ± SD	26.2 ± 3.5	12.8 ± 1.1	12.9 ± 1.1
Average time between scans (years) ± SD	>1 week	1.00 ± 0.11	1.07 ± 0.17
Race			
Black or African American	0	10	1
White	12	57	19
Native Hawaiian or Pacific Islander	0	1	0
More than one race	0	0	1
Ethnicity			
Hispanic or Latino	0	53	20
Non‐Hispanic or Non‐Latino	12	15	1

Reproducibility measures for the 12 young adults are shown in Table 2. Overall, reproducibility was excellent with MCV of 3.86% or less and MAD of 4.85% or less across three visits. The splenium of the corpus callosum followed by the whole‐brain average and thalamic radiation had the lowest MCVs of 0.76%, 0.86%, and 0.91%, respectively. The lowest MADs also occurred in these same tracts (splenium, whole‐brain average, and thalamic radiation) with differences of 0.96%, 0.98%, and 1.14%, respectively. The regions with the highest MCV and MAD were the fornix and the superior frontal‐occipital tracts, but these metrics were still under 5%. The regional ICC values in the adults were high (between = 0.91 and 0.98) for all tracts with exception of fornix (ICC = 0.77).

**Table 2:** Summary of reproducibility measures from FH+ and FH− adolescents and healthy adults

	Adults			All			FH+			FH−		
	MCV	MAD	ICC	MCV	MAD	ICC	MCV	MAD	ICC	MCV	MAD	ICC
Whole brain average	0.86	1.09	0.98	1.65	2.34	0.75	1.79	2.54	0.72	1.20	1.69	0.67
Genu	0.93	1.18	0.98	1.94	2.65	0.66	1.94	2.68	0.59	1.93	2.54	0.72
Body	1.77	2.24	0.94	2.49	3.57	0.82	2.48	3.57	0.84	2.52	3.57	0.71
Splenium	0.76	0.96	0.97	1.31	1.84	0.81	1.34	1.89	0.83	1.21	1.68	0.64
Fornix	3.86	4.85	0.77	5.42	7.25	0.62	5.33	7.14	0.64	5.73	7.63	0.52
Corticospinal	1.85	2.39	0.95	3.07	4.14	0.63	2.85	3.90	0.72	3.78	4.93	0.26
Internal capsule	1.13	1.44	0.96	2.36	3.26	0.72	2.28	3.16	0.70	2.60	3.58	0.83
Corona radiata	1.15	1.46	0.98	1.55	2.18	0.87	1.64	2.30	0.86	1.28	1.79	0.85
Thalamic radiation	0.91	1.14	0.98	1.89	2.62	0.76	1.87	2.62	0.77	1.93	2.61	0.70
Sagittal striatum	1.11	1.42	0.99	2.47	3.37	0.69	2.29	3.17	0.72	3.06	4.02	0.64
External capsule	1.28	1.62	0.97	2.50	3.45	0.75	2.38	3.29	0.76	2.88	3.95	0.80
Cingulum	1.30	1.63	0.98	2.49	3.50	0.79	2.65	3.73	0.77	1.97	2.75	0.85
Superior longitudinal fasciculus	1.10	1.40	0.98	1.79	2.51	0.87	1.85	2.61	0.87	1.58	2.21	0.88
Fronto‐occipital	1.71	2.22	0.95	1.80	2.51	0.92	1.73	2.42	0.92	2.02	2.79	0.90
Superior fronto‐occipital	2.46	3.19	0.91	2.50	3.51	0.83	2.34	3.32	0.85	3.02	4.13	0.86
Inferior fronto‐occipital	2.02	2.55	0.94	1.72	2.40	0.95	1.73	2.41	0.96	1.69	2.36	0.94
Anterior corona radiata	1.40	1.80	0.98	1.54	2.17	0.90	1.51	2.15	0.88	1.62	2.25	0.90
Superior corona radiata	1.34	1.72	0.97	2.07	2.92	0.83	2.16	3.04	0.84	1.76	2.50	0.74
Posterior corona radiata	0.94	1.22	0.98	2.22	3.11	0.86	2.31	3.25	0.85	1.90	2.65	0.84

**Figure 1:**
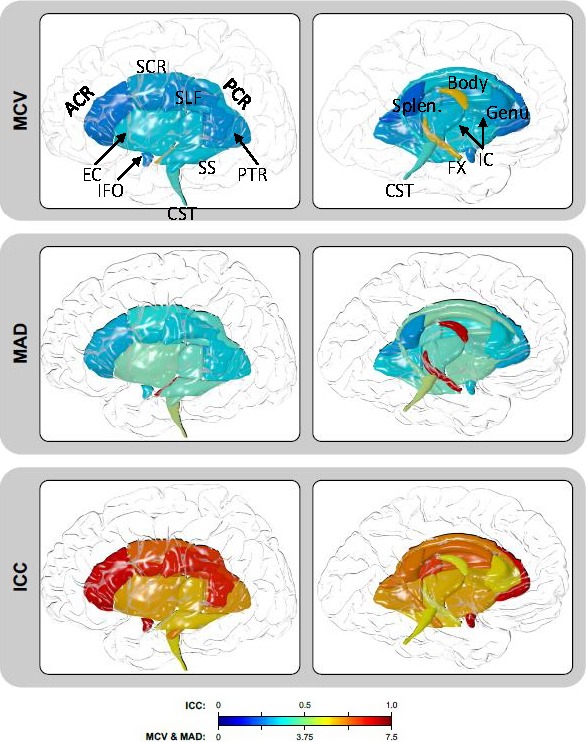
Three reproducibility measurements show the regional course of the major white matter tracts. Overall, reproducibility was excellent for most white matter regions with very low MCV and MAD values that indicate excellent reproducibility. MCV and MAD were higher as for the CST and fornix as indicated in a more intense/red color. For ICC where excellent reproducibility is reflected with a value close to 1, again reproducibility was excellent overall with ICCs close to 1. MCV, mean coefficients of variation; MAD, mean absolute difference, and ICC, intraclass correlation

**Figure 2:**
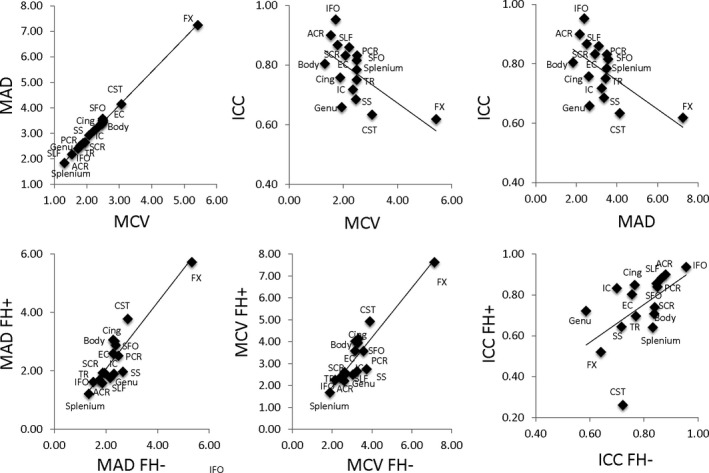
Scatter plots of the three reproducibility parameters (MAD, MCV, and ICC) for each WM tract shown with linear regression fits ranging from *r* ^2^ of 0.4–0.99. (top row). Each WM tract data point represents either MAD or MCV from both (FH+ and FH−) groups as summarized in the “All” section of Table 2. Scatter plots of the reproducibility parameters from each WM tract separated by group: youths with family histories of substance use disorders (FH+) vs. youths with no such histories (FH−; bottom row) shown with linear regression fits ranging from *r* ^2^ of 0.34–0.80. Actual MCV and MAD values for each WM tract from each group (FH+ and FH−) are summarized in Table 2

**Figure 3:**
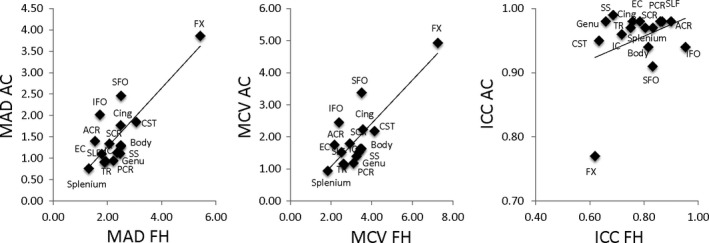
Reproducibility parameters for adult controls (AC) were plotted vs. corresponding parameters in the adolescent cohort (FH). Linear regression analysis showed high correlation between MAD and MCV measurements for individual tracts (*r *= .82, *p *< .01). The correlation between ICC measurements was not significant (*r *= .33, *p *= .2)

The mean coefficients of variation (MCV) for the average FA across the full skeleton in FH+ and FH− youths combined was 1.65% (Table 2). Regionally, the lowest MCV values were observed for the splenium of the corpus callosum at 1.31%, followed by anterior corona radiata (ACR; Figure 1, Table 2). Highest year‐to‐year variances were observed for the fornix (5.42%) and corticospinal tracts (CST, 3.07%). The trends in mean absolute differences (MAD) mirrored the trends in MCV (*r *= .95). The splenium and ACR had the lowest MAD values and the fornix and corticospinal tracts having the highest MAD values (Figure 2, top row). The ICC for the average FA values was 0.75. The highest ICC was observed for inferior fronto‐occipital tract, followed by fronto‐occipital tract (ICC = 0.95 and 0.92, respectively). The lowest ICCs were observed for the fornix and CST (ICC = 0.62 and 0.63, respectively). Overall, the MCV and MAD measurements were higher (3.86% or more and 4.85% or more, for MCV and MAD, respectively) in this group than in adults. The adult and adolescent groups showed an excellent agreement for regional MCV and MAD metrics (Figure 3). Despite good agreement between regional MCV and MAD patterns, the regional ICC pattern in this sample was not significantly correlated with the adult cohort (*r *=* *.28, *p *>* *.2).

Similar patterns of variance were observed when FH+ and FH− youths were examined separately (Figure 2, bottom row). Again, the splenium and ACR tracts had the lowest MCV and MAD values in the FH+ group, whereas the splenium and corona radiata had the lowest in the FH− group. The fornix and CST had the highest MCV and MAD values in both groups. Statistical analyses revealed that there were no significant group‐wise differences in MCV or MAD values after correction for multiple comparisons. In terms of ICC, the inferior fronto‐occipital tract had the largest ICC in both groups.

One‐way ANOVAs performed on tract‐wise MCV, MAD, and ICC values showed no significant differences (*p *>* *.2). Linear correlation analyses for the tract‐wise MCV and MAD values between FH+ and FH− groups showed excellent agreement between tracts with *r *=* *.837 and .841. Likewise, linear regression analyses between adults and all adolescents (both FH+ and FH−) showed good agreement between tracts with *r* of .66 and .63, respectively (*p *=* *.01). The correlation between adults and adolescents (FH+ and FH−) was not significant (*r *= .1, *p *> .5). Correlation analyses of tract ICC metrics between groups showed significant, albeit lower correlation (*r *=* *.57, *p *=* *.03) for FH+ and FH−.

**Figure 4:**
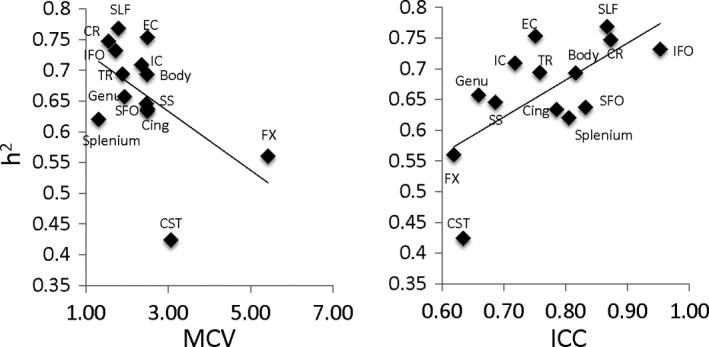
Heritability (*h* ^2^) measurements for regional FA values calculated by ENIMGA‐DTI workgroup were plotted versus MCV and ICC reproducibility parameters in the adolescent cohort (FH). Linear regression analysis showed a significant and negative correlation between *h* ^2^ and CV (*r *= −.53, *p *= .04) and a significant positive correlation with ICC (*r *= −.65, *p *= .01). The plot for *h* ^2^ and MAD values was identical to that of MCV and therefore was omitted

Finally, the similarity between the regional patterns, our reproducibility results, and the heritability results presented in previous ENIGMA‐DTI genetic work (Jahanshad et al., 2013; Kochunov et al., 2015; Kochunov et al., 2015a,b) motivated a post hoc comparison of reproducibility measures on the heritability. Here, reproducibility measurements explained between 25% and 49% of the variance in the tract‐wise estimates of heritability (*h* ^2^) reported by ENIGMA‐DTI (Kochunov et al., 2015) (Figure 4). The highest correlation was observed between *h* ^2^ and ICC (*r *= .65, *p *= .01, Figure 4). The correlation between *h* ^2^ and MCV and MAD were both negative (*r *= −.53, *p *= .01) (Figure 4).

## 4 Discussion

We examined reproducibility of FA values provided by the ENIGMA‐DTI protocol in 12 healthy adults, scanned three times within 1‐week period, where no changes in white matter microstructure were expected. Parallel analyses were performed in a large cohort of adolescents imaged twice, 1‐year apart, where only modest developmental changes in white matter microstructure were expected. ENIGMA‐DTI protocol is unique in that it was specifically developed and validated to provide homogenized measurements of DTI parameters across diverse population samples and imaging protocols for genetic imaging analyses (Jahanshad et al., 2013). This protocol was shown to provide a regionally consistent pattern of heritability in several large population studies (Kochunov et al., 2013, 2001). Here, we assessed the reproducibility of this protocol with three standard metrics: mean absolute difference (MAD), mean coefficients of variation (MCV), and intraclass correlations (ICC). In adult subjects, reproducibility was excellent with MCV of 3.86% or less and MAD of 4.85% or less for all tracts. Except for the fornix with an ICC of 0.77, all other tracts had ICCs of 0.91 or better. Thus, the adult cohort established the excellent reproducibility of the technique and served as a comparison to our adolescent cohort that showed low variance between time points, which is expected since changes in white matter microstructure are small compared to between subject variance. In the adolescents, the largest variance was observed for the corticospinal tract (CST) and fornix (FX) area. Poor reproducibility of the CST and FX was also observed in the adult cohort, suggesting that the low reproducibility in these tracts is caused by methodological confounds rather than a consequence of adolescent development. The regional pattern of reproducibility measurements was similar to the variance in the heritability measurements reported by the ENIGMA‐DTI group (Kochunov et al., 2015, 2015b) driven primarily by low heritability estimates in these two tracts. Overall, our analyses demonstrated excellent reproducibility of ENIGMA‐DTI FA measurements, positing this protocol as a viable analysis tool for longitudinal studies of white matter microstructural development. Future work evaluating these regions should take caution in interpreting any results localized to the CST and FX.

The findings of reduced reproducibility of the FX and CST were likely caused by two known methodological limitations: partial voluming effects and/or spatial misregistration. Both limitations can lead to errors during projection of FA values on the group‐wise skeleton. The FX and CST are narrow, tubular white matter structures located in the areas with susceptibility for geometrical distortions (Bach et al., 2014). The spatial course of the FX parallels that of the *stria terminalis*, and the projection step may randomly fail to separate these tracts (Vasung et al., 2012). The CST is particularly susceptible to misregistration errors due to its spatial course and variability in the field of view of the image (Bach et al., 2014). The CST extends down to the level of mesencephalon and medulla where the combination of long echo time and presence of susceptibility can cause geometrical distortions that depend on head orientation and shimming parameters of MRI acquisition. Poor reproducibility for CST FA measurements was also reported in a study by Bisdas and colleagues (Bisdas, Bohning, Besenski, Nicholas, & Rumboldt, 2008).

Overall, the reproducibility of FA values extracted with ENIGMA‐DTI protocol was comparable or superior to the measurements reported by other reproducibility studies of FA. Jansen and colleagues limited their analyses to voxel‐wise FA values for frontal and temporal lobes and the cerebrum from 10 healthy adults imaged twice over a 1‐month period and reported MCVs ranging from 3.0% to 5.3%, which were slightly worse than the estimates obtained in our study (Jansen, Kooi, Kessels, Nicolay, & Backes, 2007). The reported ICCs (0.46–0.73) were also lower than that observed in our study (0.77–0.98). Differences in MCV between this study and our study may be due to differences in acquisition parameters such as number of gradient directions and voxel sizes. Veenith and colleagues scanned 22 healthy adults three times to generate within session and between session reproducibility metrics (Veenith et al., 2010). That study reported comparable reproducibility via MCVs for major tracts such as the genu, body, and splenium of the corpus callosum as well as the cingulum and longitudinal fasciculus. Grech‐Sollars et al. (2015), Teipel et al. (2015), and Vollmar et al. (2013) examined reproducibility of DTI measures collected in participants scanned on multiple scanners (Grech‐Sollars et al., 2015; Teipel et al., 2015; Vollmar et al., 2013). Our results are similar to those of the Grech‐Sollars and colleagues study that reported a intrascanner, intravolunteer CV of 2.1%, and a intervolunteer, intrascanner CV of 3.8% for white matter overall and those of Vollmar et al. (2013) showing MCV of the splenium of the corpus callosum at 2% or less and an ICC of 0.9 or greater. Teipel and colleagues showed that track‐based statistics was superior to deformation‐based analyses in terms of reproducibility with an overall mean CV of 14% across 14 scanners (Teipel et al., 2011). When reducing the analyses to calculate MCV between two scanners, the MCV for the splenium of the corpus callosum was 3.4%, slightly higher than was observed here, but still very good. A study by Jovicich et al. (1999) quantified reproducibility of FA values at multiple imaging sites where five adults (age range: 50–80) were scanned per site over a 2‐month period. Overall, our study showed excellent reproducibility for our FA measurements.

We found no significant differences in MCV, MAD, or ICC among participants with and without family histories of alcohol and other substance use disorders. The lack of group differences indicates that the FA differences between the groups (Table S1) reflect differences in underlying white matter microstructure rather than driven by methodological issues. Furthermore, the high reproducibility of the adult population global and regional FA metrics indicates this protocol will likely be sensitive to changes occurring as a result of normal adolescent development as well as potential neuroanatomical changes that may develop as a result of problem substance use.

Reproducibility measurements obtained by our study were predictive of the heritability values reported by ENIGMA‐DTI Working Group (Kochunov et al., 2015, 2015b). The significant correlation for all three parameters suggested that regional FA measurements with lower reproducibility tend to show reduced heritability estimates. The heritability estimate is the proportion of the total variance in the trait that can be explained by additive genetic factors; this is often estimated using known genetic relationships between family members, including those in large pedigrees or twin‐sibling studies. Heritability values are sensitive to presence of random or methodologically related variability that may inflate the total variance and reduce the proportion that is attributable to additive genetic variance. This study confirms that the lower heritability values for CST and fornix tracts reported by ENIGMA‐DTI were driven by limitations of the method. Any findings regarding FA measurements for these tracts should be considered with caution.

## 5 Conclusions

In summary, the ENIGMA‐DTI protocol yields highly reproducible tract‐based FA values, indicating that this protocol is suitable for monitoring white matter microstructural changes over time in longitudinal samples using the same DTI acquisition protocol. In our ongoing work, we will be using this protocol to monitor white matter microstructural changes in our cohort of FH+ and FH− youths due to normal development as well as changes resulting from the initiation and progression of substance use.
