# Supplementary figures and images for: Reproducibility of tract‐based white matter microstructural measures using the ENIGMA‐DTI protocol
Source: Brain Behav. 2017 Jan 14;7(2):e00615. doi: 10.1002/brb3.615 (PMC5318368; doi:10.1002/brb3.615)

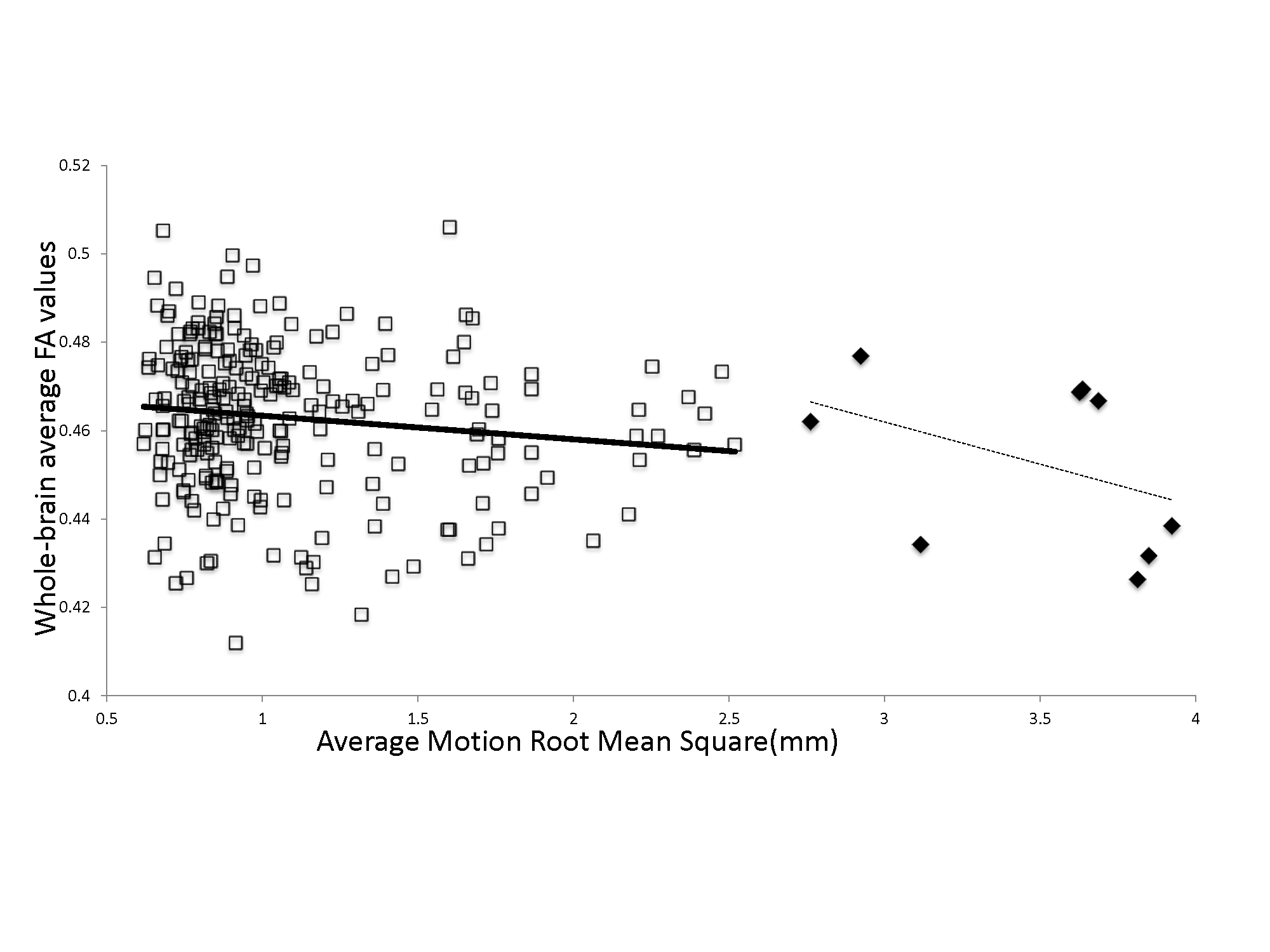

Supplement: Supplementary file 1 [file BRB3-7-e00615-s001.tiff]

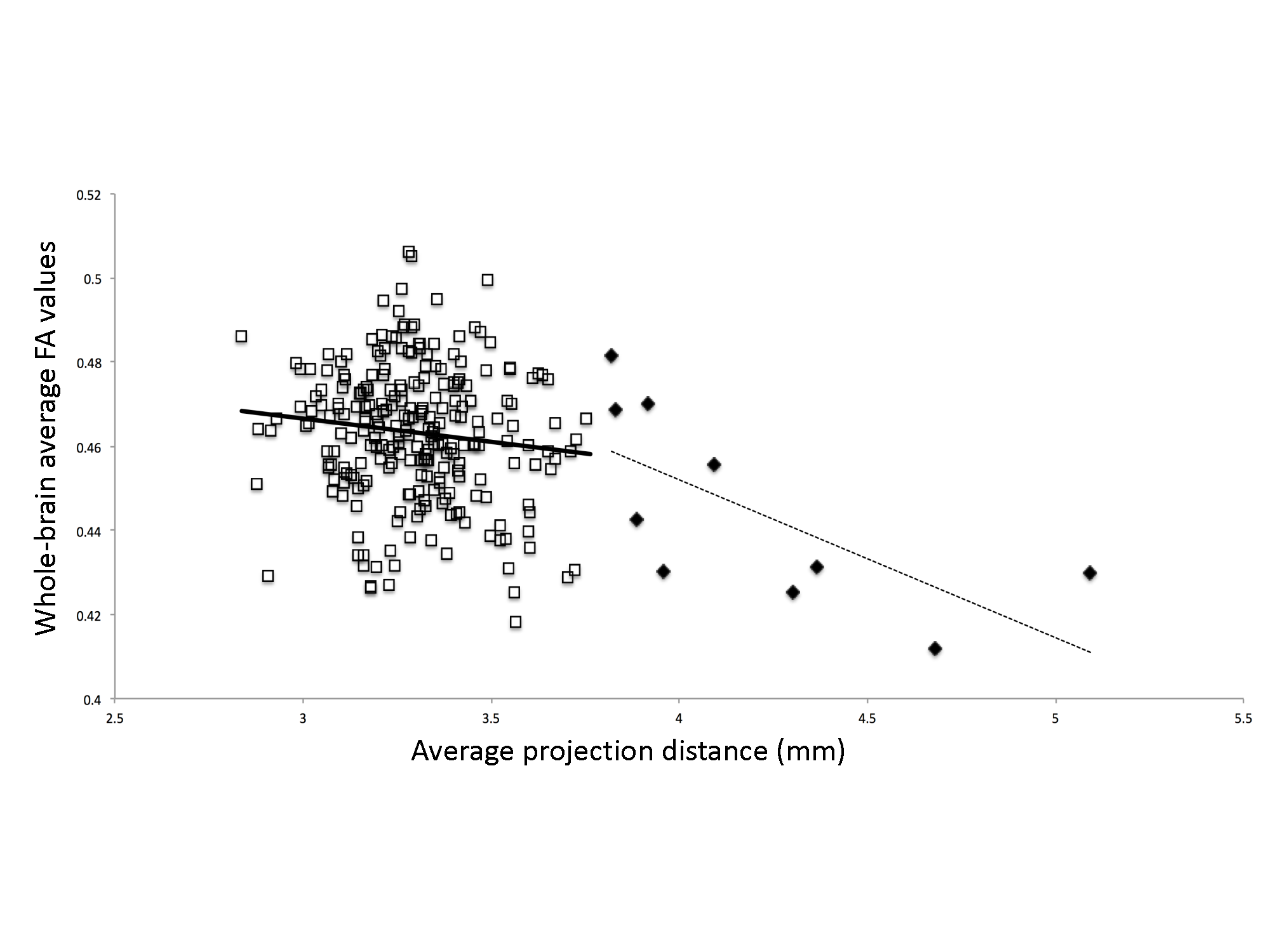

Supplement: Supplementary file 2 [file BRB3-7-e00615-s002.tiff]
