# Supplementary material for: Reproducibility of tract‐based white matter microstructural measures using the ENIGMA‐DTI protocol
Source: Brain Behav. 2017 Jan 14;7(2):e00615. doi: 10.1002/brb3.615 (PMC5318368; doi:10.1002/brb3.615)
Supplement: Supplementary file 3 [file BRB3-7-e00615-s003.docx]

| Supplementary Table 1. Summary of Mean FA values for Adults and FH+ and FH- groups | | | | | | | |
| --- | --- | --- | --- | --- | --- | --- | --- |
|  |  | Adults | | FH+ | | FH- | |
|  | Visit | Mean | Stdev | Mean | Stdev | Mean | Stdev |
| Average FA | 1 | 0.497 | 0.019 | 0.459 | 0.015 | 0.473 | 0.009 |
|  | 2 | 0.497 | 0.023 | 0.461 | 0.016 | 0.471 | 0.016 |
|  | 3 | 0.498 | 0.022 |  |  |  |  |
| Genu | 1 | 0.747 | 0.031 | 0.745 | 0.028 | 0.772 | 0.029 |
|  | 2 | 0.744 | 0.035 | 0.747 | 0.041 | 0.762 | 0.062 |
|  | 3 | 0.746 | 0.037 |  |  |  |  |
| Body | 1 | 0.720 | 0.035 | 0.656 | 0.039 | 0.672 | 0.027 |
|  | 2 | 0.712 | 0.039 | 0.668 | 0.038 | 0.677 | 0.043 |
|  | 3 | 0.718 | 0.048 |  |  |  |  |
| Splenium | 1 | 0.831 | 0.029 | 0.764 | 0.024 | 0.776 | 0.017 |
|  | 2 | 0.829 | 0.031 | 0.763 | 0.027 | 0.772 | 0.029 |
|  | 3 | 0.831 | 0.032 |  |  |  |  |
| Fornix (FX) | 1 | 0.535 | 0.026 | 0.478 | 0.033 | 0.483 | 0.027 |
|  | 2 | 0.534 | 0.031 | 0.458 | 0.047 | 0.450 | 0.034 |
|  | 3 | 0.536 | 0.046 |  |  |  |  |
| Corticospinal (CST) | 1 | 0.701 | 0.043 | 0.550 | 0.033 | 0.561 | 0.025 |
|  | 2 | 0.703 | 0.046 | 0.545 | 0.043 | 0.537 | 0.043 |
|  | 3 | 0.702 | 0.037 |  |  |  |  |
| Internal capsule (IC) | 1 | 0.680 | 0.026 | 0.625 | 0.020 | 0.636 | 0.016 |
|  | 2 | 0.679 | 0.030 | 0.613 | 0.027 | 0.614 | 0.024 |
|  | 3 | 0.683 | 0.032 |  |  |  |  |
| Corona radiata (CR) | 1 | 0.529 | 0.025 | 0.495 | 0.018 | 0.509 | 0.014 |
|  | 2 | 0.528 | 0.026 | 0.493 | 0.021 | 0.505 | 0.017 |
|  | 3 | 0.531 | 0.028 |  |  |  |  |
| Thalamic radiation (TR) | 1 | 0.667 | 0.026 | 0.625 | 0.027 | 0.641 | 0.022 |
|  | 2 | 0.666 | 0.032 | 0.622 | 0.027 | 0.631 | 0.039 |
|  | 3 | 0.665 | 0.031 |  |  |  |  |
| Sagittal striatum (SS) | 1 | 0.607 | 0.040 | 0.565 | 0.026 | 0.578 | 0.026 |
|  | 2 | 0.606 | 0.045 | 0.561 | 0.032 | 0.562 | 0.049 |
|  | 3 | 0.609 | 0.042 |  |  |  |  |
| External capsule (EX) | 1 | 0.538 | 0.024 | 0.489 | 0.019 | 0.496 | 0.016 |
|  | 2 | 0.539 | 0.029 | 0.481 | 0.024 | 0.477 | 0.021 |
|  | 3 | 0.541 | 0.027 |  |  |  |  |
| Cingulum (Cing) | 1 | 0.682 | 0.037 | 0.611 | 0.031 | 0.626 | 0.023 |
|  | 2 | 0.680 | 0.037 | 0.607 | 0.035 | 0.616 | 0.031 |
|  | 3 | 0.685 | 0.043 |  |  |  |  |

| Superior longitudinal fasciculus (SLF) | 1 | 0.550 | 0.029 | 0.0506 | 0.024 | 0.519 | 0.018 |
| --- | --- | --- | --- | --- | --- | --- | --- |
|  | 2 | 0.549 | 0.031 | 0.504 | 0.024 | 0.510 | 0.017 |
|  | 3 | 0.551 | 0.031 |  |  |  |  |
| Superior fronto-occipital (SFO) | 1 | 0.592 | 0.035 | 0.525 | 0.026 | 0.547 | 0.025 |
|  | 2 | 0.591 | 0.032 | 0.524 | 0.035 | 0.526 | 0.029 |
|  | 3 | 0.593 | 0.037 |  |  |  |  |
| Inferior fronto-occipital (IFO) | 1 | 0.593 | 0.034 | 0.521 | 0.042 | 0.529 | 0.036 |
|  | 2 | 0.587 | 0.037 | 0.516 | 0.043 | 0.525 | 0.037 |
|  | 3 | 0.599 | 0.040 |  |  |  |  |
| Anterior corona radiata (ACR) | 1 | 0.502 | 0.028 | 0.464 | 0.021 | 0.480 | 0.021 |
|  | 2 | 0.502 | 0.029 | 0.464 | 0.020 | 0.477 | 0.021 |
|  | 3 | 0.506 | 0.031 |  |  |  |  |
| Superior corona radiata (SCR) | 1 | 0.537 | 0.025 | 0.505 | 0.023 | 0.517 | 0.017 |
|  | 2 | 0.536 | 0.028 | 0.503 | 0.029 | 0.515 | 0.025 |
|  | 3 | 0.539 | 0.027 |  |  |  |  |
| Posterior corona radiata (PCR) | 1 | 0.548 | 0.026 | 0.515 | 0.024 | 0.531 | 0.017 |
|  | 2 | 0.546 | 0.024 | 0.510 | 0.045 | 0.523 | 0.024 |
|  | 3 | 0.548 | 0.028 |  |  |  |  |
